# Phasic Contractions in Urinary Bladder from Juvenile versus Adult Pigs

**DOI:** 10.1371/journal.pone.0058611

**Published:** 2013-03-13

**Authors:** Bahareh Vahabi, Donna J. Sellers, Dominika A. Bijos, Marcus J. Drake

**Affiliations:** 1 Department of Applied Sciences, University of the West of England, Bristol, United Kingdom; 2 Bristol Urological Institute, Southmead Hospital, Bristol, United Kingdom; 3 Faculty of Health Sciences and Medicine, Bond University, Queensland, Australia; 4 Schools of Clinical Sciences, University of Bristol, Bristol, United Kingdom; UCL Institute of Child Health, United Kingdom

## Abstract

**Aims:**

Alterations in properties of the bladder with maturation are relevant physiologically and pathophysiologically. The aim of this study was to investigate alterations in bladder properties with maturation in juvenile vs. adult pig, focussing on differences between layers of the bladder wall (mucosa vs. detrusor) and the presence and functional contribution of interstitial cells (ICs).

**Methods:**

Basal and cholinergic-induced phasic contractions (PCs) in mucosal and denuded-detrusor strips from juvenile and adult pigs were assessed. Expression of c-kit, a marker of ICs, was investigated in the mucosa and the detrusor layers of the pig bladder. The functional role of ICs in mediating PCs was examined using imatinib.

**Results:**

Mucosal strips from juvenile and adult pig bladders demonstrated basal PCs whilst denuded-detrusor strips did not. PCs of mucosal strips from juvenile pigs were significantly greater than those from adult bladders. Immunoreactivity for c-kit was detected in mucosa and detrusor layers of pig bladder. Histological studies demonstrated a distinct layer of smooth muscle between the urothelium and bladder detrusor, termed the muscularis mucosa. Imatinib was only effective in inhibiting PCs in mucosal strips from juvenile pigs. Imatinib inhibited the carbachol-induced PCs of both juvenile and adult denuded-detrusor strips, although strips from juvenile bladders demonstrated a trend towards being more sensitive to this inhibition.

**Conclusions:**

We confirm the presence of c-kit positive ICs in pig urinary bladder. The enhanced PCs of mucosal strips from juvenile animals could be due to altered properties of ICs or the muscularis mucosa in the bladders of these animals.

## Introduction

Normal bladder function is complex, resulting from co-operative interaction of numerous functional cell types. The detrusor muscle has been extensively characterized and is responsible for the majority of the contractile properties of the bladder, whilst urothelium has recently been recognised to possess a broad range of sensory and signal transduction functions that greatly influence bladder contractility and physiology [1]. The detrusor exhibits nerve-evoked and spontaneous phasic contractions (PCs) which are regulated by the release of neurotransmitters (such as acetylcholine (ACh) and ATP) and spontaneous action potentials (through activation of calcium channels) respectively [2]. Recent studies have shown that the urothelium and lamina propria from the bladder also display spontaneous and agonist-induced contractile activity [3], [4]. In addition, it has been reported that the spontaneous contractions of the detrusor might be linked to the urothelium and the lamina propria [5]. However, the region between the basement layer of the bladder urothelium and the luminal surface of the detrusor muscle (lamina propria) has a complicated structure and contains sensory nerves, a dense network of capillaries, a diverse group of cells, including interstitial cells (ICs), and a type of smooth muscle termed the muscularis mucosa [6], [7]. Therefore, it is unclear which cell types mediate the spontaneous bladder contractions, although it has been suggested that ICs may be involved [3], [4].

Studies have demonstrated differences in contractile properties of the detrusor from young and adult animals [8], [9], [10]. Significant differences in the phasic activity of bladder strips from neonatal vs. adult rats have been reported [11], [12]. Neonatal rat bladders demonstrate high amplitude, rhythmic contractions which may promote voiding, since neural control is still immature [9]. These rhythmic contractions are believed to originate from urothelium-suburothelium near the bladder dome and are enhanced by stretch and carbachol, leading to the hypothesis that ACh is released from the urothelium during bladder filling to enhance spontaneous activity [9]. Therefore, altered bladder properties with maturation are relevant physiologically and may signify potential pathophysiological mechanisms. Very few studies have looked at the contractile properties of the pig bladder, considered a good model for human bladder function [13], [14], during maturation. Wuest et al (2005) demonstrated larger atropine-resistant contractions in juvenile (6–8 weeks) vs. adult (40 weeks) pig bladders, suggesting a strong component of non-cholinergic contraction in juvenile tissues [15]. Accordingly, the present study evaluated: 1) the properties of juvenile and adult pig bladders, examining differences between layers of the bladder wall (mucosa versus detrusor) with maturation and 2) the presence and functional contribution of c-kit positive ICs using a pharmacological mediator of these cells, imatinib (Glivec®; Novartis Pharmaceuticals), a non-selective inhibitor of the tyrosine kinase activity of c-kit.

## Methods

### Tissue Preparation

Female pig (*Sus scrofa domestica*, ∼6-weeks and ∼6-months old) bladders obtained from a local abattoir (with permission of Langford abattoir, University of Bristol, Bristol, UK) were placed in cold Krebs bicarbonate solution (mM: NaCl 118.4, NaHCO_3_ 24.9, KCl 4.7, CaCl_2_ 1.9, MgSO_4_ 1.15, KH_2_PO_4_ 1.15, glucose 11.7). From each bladder dome, two sets of longitudinally orientated strips (2×10 mm^2^) of denuded-detrusor and mucosa-only (consisting of the urothelium, lamina propria and muscularis mucosa) were dissected. Mucosal strips were prepared following the natural plane of division. For denuded detrusor strips, individual muscle bundles with good longitudinal alignment were dissected along their line of orientation using magnified visual inspection. Tissue strips were mounted in Perspex microbaths (volume 0.2 mL, Oxford University, UK) and were superfused with carboxygenated Krebs at a flow rate of 1.8±0.3 mL·min^−1^ via a peristaltic pump. The circuit passed through a heated bath which maintained the strips at 36±0.5°C.

### Functional Studies

Strips were equilibrated under a resting tension of 1.5–2 g for 60 min, as this lies in the range of maximum active tension generation for passively tensioned muscle strips [16]. Tension was monitored via isometric force transducers (Pioden Controls Ltd, UK) and a Powerlab data acquisition system running ‘Chart’ software (ADInstruments, UK). An initial test response was elicited in all mucosal and denuded-detrusor strips from both juvenile and adult bladders by exposure for 10-seconds to carbachol (CCh), dissolved in Krebs solution (10 µM). The tonic contractile response to 10 µM CCh was calculated in all tissue strips. After a 15 min washout period, mucosal strips were perfused with increasing concentrations of imatinib (1–50 µM; 15–20 min exposure to each concentration; stock solution dissolved in Krebs). Denuded-detrusor strips were constantly superperfused with 0.1 µM CCh to induce phasic activity, as previously described [17]. In the continuous presence of CCh, denuded-detrusor strips were then perfused with increasing concentrations of imatinib (1–50 µM). The effect of imatinib on the amplitude and the frequency of basal PCs in mucosal strips and cholinergic-induced PCs in denuded-detrusor strips was assessed.

The mean amplitude/mg tissue and frequency of PCs during a 5 min period, within the 10–15 min drug exposure period, were calculated and data expressed as mean±SEM. To calculate the amplitude and the frequency of PCs, a method slightly modified from that proposed by Imai *et al* (2001) [18], as used in our previous studies [17] was used to define a single spontaneous contractile event. Firstly, the average peak amplitude of contractions over a 5 min period was calculated, with the minimum zero force being the preload tension prior to the start of the phasic contractions. For frequency calculations, any contractions over and above 30% of this average peak amplitude were considered as single spontaneous contractions. Where a contraction was superimposed on the previous event before reaching baseline, the two contractions were considered a single contraction event [18]. For statistical analysis a repeated measures ANOVA with Dunnett’s post hoc test and unpaired Student’s t-test were used for intra- and inter-tissue variations respectively. P<0.05 was considered statistically significant.

### Molecular Studies

Total RNA was extracted from denuded-detrusor and mucosa from juvenile (n = 3) and adult (n = 3) pig bladders using a Ribo PureTM kit (Ambion, Warrington, UK) according to manufacturer’s instructions, including the removal of trace genomic DNA. Total RNA (300–500 ng/ µL) was reverse transcribed to single-strand cDNA using Superscript III enzyme along with oligo dT primers as per manufacturer’s instruction (Invitrogen, UK). cDNA samples were first amplified using GAPDH forward (5′ACCCAGAAGACTGTGGATGG 3′) and reverse (5′CACATTGGGGGTAGGAACAC3′) primers to confirm successful reverse transcription (positive control). Sterile water reaction was included instead of cDNA template as negative control. Following confirmation of the presence of amplifiable cDNA, samples were amplified using primers specific to c-kit (accession no: FJ938289.1), forward (5′GGTCTAGCCAGAGACATCAG 3′) and reverse (5′CAGCGGCTGCGTGGAGGAGG 3′) primers. All primers were supplied by Invitrogen, UK.

The amplification conditions consisted of denaturation at 95°C for 5 min, 35 cycles of 95°C for 15 sec, 59°C for 1 min and 70°C for 1 min, and a final extension at 70°C for 10 min. Amplified PCR products were separated by agarose gel electrophoresis to confirm successful amplification, purified using QIAquick Gel (QIAGEN, UK) extraction kit and sent to GATC-Biotech (London, UK) for direct sequencing. The sequenced data was then verified by comparison with genome database using BLAST from NCBI website (http://blast.ncbi.nlm.nih.gov/blast.cgi).

### Immunohistochemistry

Sections of fresh intact bladder dome, mucosal and denuded-detrusor strips from adult and juvenile pigs were fixed (4% neutral buffered formalin), processed and embedded in paraffin. Tissues were sectioned at 3 µm and placed on charged slides. Some sections were stained using haematoxylin and eosin with others processed for immunohistochemistry using antibodies directed at c-kit and vimentin. Following removal, endogenous peroxidase activity was blocked in 0.3% hydrogen peroxide for 10 min. Slides were placed in 0.1 M sodium citrate antigen retrieval buffer and heated in a 750 W microwave oven for 20 min, followed by incubation with c-kit rabbit polyclonal antibody (1∶250) or vimentin mouse monoclonal antibody (1∶500) overnight at 4°C. Detection used an avidin biotin peroxidase system (Vector Laboratories Ltd. UK) and a diaminobenzidine tetrahydrochloride chromogen (Dakocytomation, UK). Negative controls replaced the primary antibody with the c-kit peptide (1∶60) or tris buffered saline. Sections were counterstained with haematoxylin and examined using a Nikon Eclipse 50i microscope (Nikon Instruments Inc., USA). All antibodies were supplied from Santa Cruz Biotechnology, Germany.

## Results

### Functional Studies

#### Tonic contractile response to 10 µM CCh in mucosal and denuded-detrusor strips

**Figure 1:**
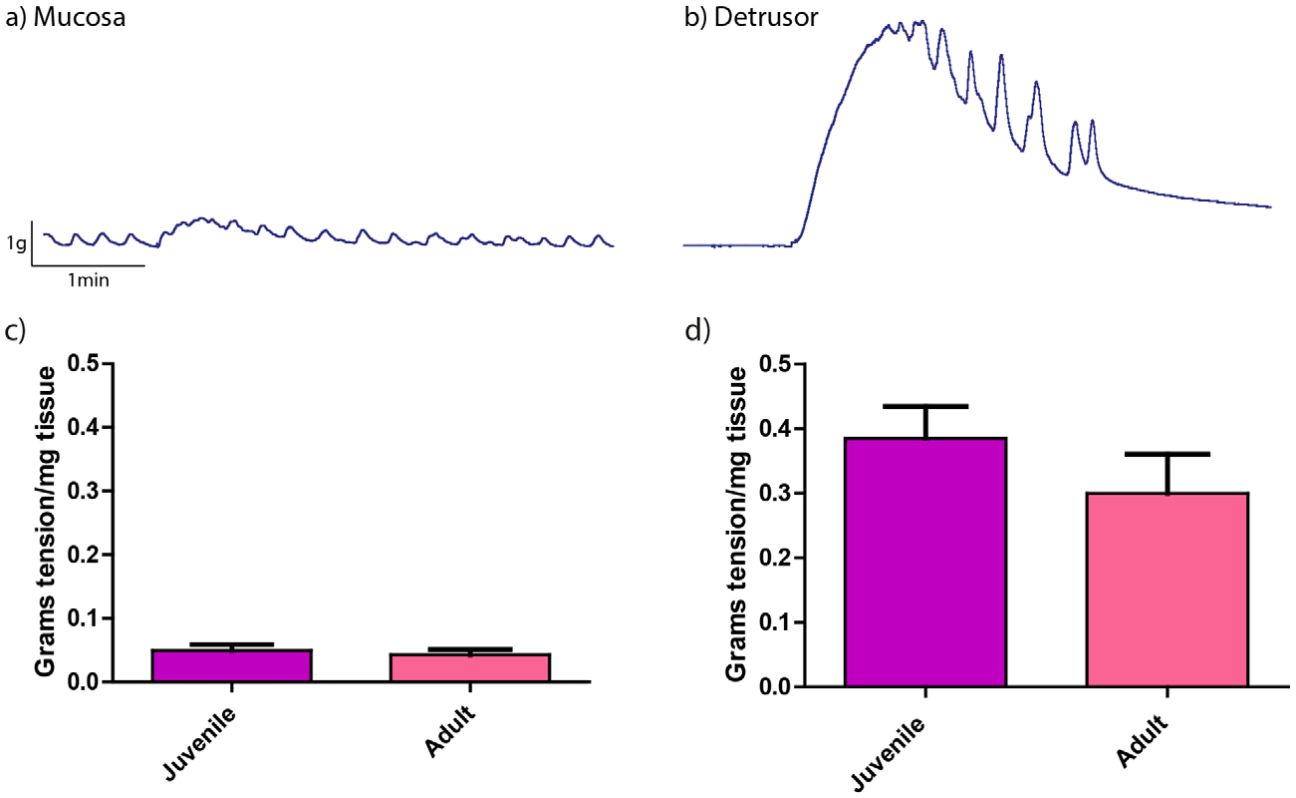
Tonic contractile responses to CCh in mucosal and denuded-detrusor strips. Tonic contractile response to CCh (10 µM) in a representative mucosal strip (a) and a denuded-detrusor strip (b) from an adult pig bladder. The mean responses to CCh were similar in mucosal strips from juvenile (n = 19) vs. adult (n = 26) pig bladders (c) and denuded detrusor strips from juvenile (n = 24) vs. adult (n = 26) pig bladders (d). Data is presented as mean±SEM.

All mucosal and denuded-detrusor strips from adult and juvenile pig bladders contracted in response to 10 µM CCh. The tonic contractile response in mucosal strips (0.05±0.01 grams tension/mg tissue in juvenile (n = 19) and 0.04±0.01 grams tension/mg in adults (n = 26)) was significantly less than denuded-detrusor strips (0.39±0.05 grams tension/mg in juvenile (n = 24) and 0.30±0.06 grams tension/mg in adult (n = 26)) in both juvenile and adult pig bladders (p<0.001). This difference could be due to a higher proportionate content of non-muscle cells in mucosal strips. However, responses to CCh in the mucosal or the denuded strips did not differ between adult and juvenile pigs (Figure 1).

#### Basal and CCh-induced PCs in mucosal and denuded-detrusor strips

**Figure 2:**
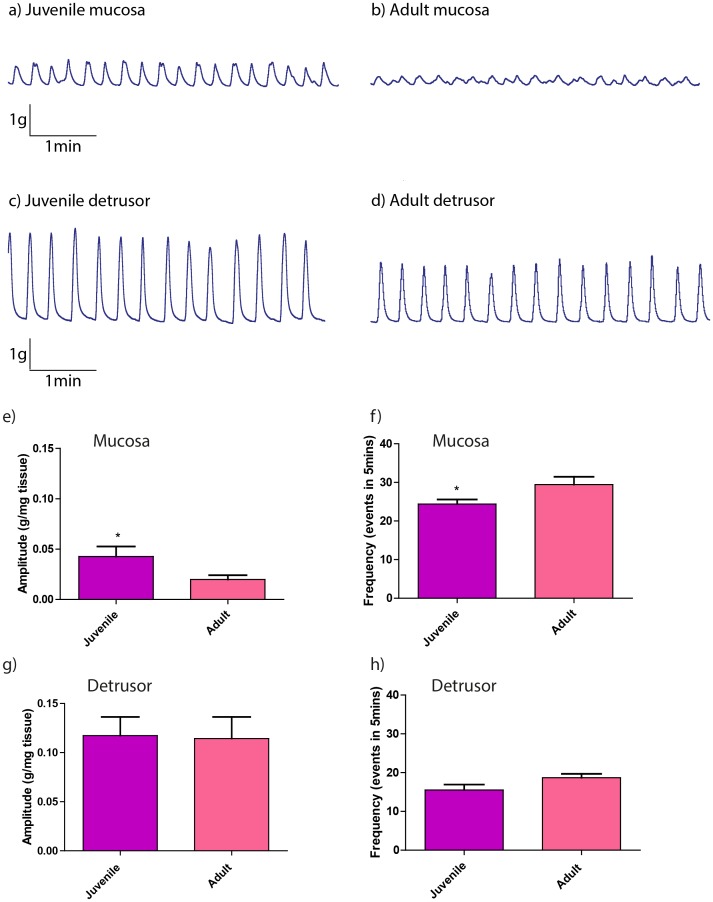
Basal and CCh-induced PCs in mucosal and denuded-detrusor strips from juvenile and adult pig bladders. Typical chart recordings of basal PCs in juvenile (a) and adult mucosal strips (b), CCh-stimulated PCs in juvenile (c) and adult (d) denuded-detrusor strips. The amplitude of basal PCs in mucosal strips from juvenile (n = 21) pigs was significantly larger than those seen in adult (n = 22) pig tissues (e). The frequency of basal PCs in juvenile mucosal strips was significantly less than those from adult pig bladders (f). Amplitude (g) and frequency (h) of CCh-stimulated PCs in denuded-detrusor strips from juvenile (n = 22) vs. adult (n = 25) pig bladders were similar. Data is presented as mean±SEM (*p<0.05, unpaired t-test).

The mucosal strips from both juvenile and adult pig bladders developed basal PCs within 20 min of equilibration; the amplitude of basal PCs in juvenile mucosal strips was significantly greater (p<0.05) than in mucosal strips from adult pigs (Figures 2a, b and e). In contrast, the frequency of basal PCs in the mucosal strips from juvenile pigs was significantly less (p<0.05) than that observed in adult tissues (Figures 2a, b and f).

Denuded-detrusor strips from juvenile and adult pig bladders did not demonstrate any basal PCs. In additional experiments, PCs were induced in denuded-detrusor strips by 0.1 µM CCh. The amplitude and frequency of CCh-induced PCs of these strips were not significantly different in juvenile vs. adult pig bladders (Figures 2c, d, g and h).

#### The effect of imatinib on basal and CCh-induced PCs in mucosal and denuded-detrusor strips

**Figure 3:**
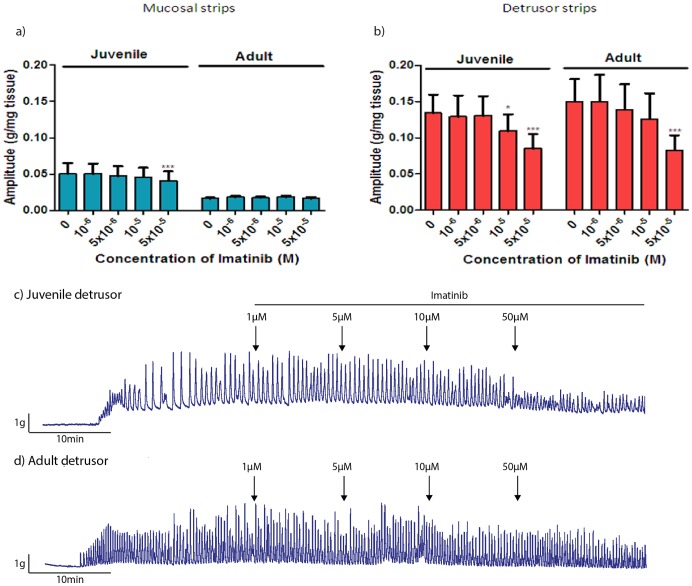
Effect of imatinib on basal and CCh-induced PCs in mucosal and denuded-detrusor strips. Effect of increasing concentration of imatinib (1–50 µM) on amplitude of basal PCs in mucosal strips (a) and CCh-induced PCs in denuded-detrusor strips (b) from juvenile (n = 13) vs. adult (n = 16) pig bladders. Typical chart recordings show the effect of increasing concentrations of imatinib on CCh-induced PCs in denuded-detrusor strips from juvenile (c) and adult (d) pig bladders. Data is presented as mean±SEM (*p<0.05, **p<0.01 vs. 0 µM imatinib, one-way ANOVA followed by Dunnett’s post hoc test).

Imatinib significantly inhibited the amplitude of basal PCs in juvenile pig bladder mucosal strips, but only at the highest concentration (50 µM; p<0.001, Figure 3a), with no significant effect on the frequency of PCs (data not shown). Imatinib did not have a significant effect on the amplitude (Figure 3a) or the frequency of basal PCs in adult mucosal strips (data not shown).

Imatinib also significantly reduced the amplitude of CCh-induced PCs of denuded-detrusor strips from juvenile pigs, but only at higher concentrations (10 µM and 50 µM; p<0.05–0.001, Figure 3b and 3c), with no significant effect on the frequency of PCs (data not shown). In denuded-detrusor strips from adult pigs imatinib significantly reduced (p<0.001) the amplitude of CCh-induced PCs only at 50 µM (Figures 3b and 3d), with no significant effect on the frequency of PCs.

The effect of vehicle (distilled water) was not significant for any tissue group (data not shown).

### Molecular Studies

**Figure 4:**
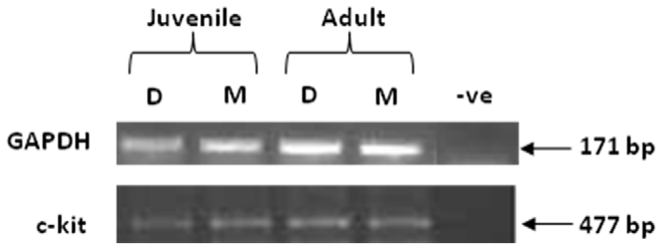
Expression of c-kit tyrosine kinase mRNA in pig bladder. Expression of c-kit mRNA (≈477 bp) was detected in detrusor (D) and mucosa (M) of juvenile and adult pig bladders; positive control was amplified with GAPDH primers (≈171 bp); negative control was amplified with water.

The presence of cDNA in mucosal and denuded-detrusor samples from juvenile and adult pig bladders was confirmed by amplification of GAPDH and observation of a band at the expected product size (∼170 bp, Figure 4). Subsequently, RT-PCR demonstrated the expression of c-kit in cDNA samples from mucosal and denuded-detrusor from both juvenile and adult pig bladders (Figure 4). Direct sequencing of the c-kit amplicons yielded partial sequences (∼300–450 bp), with 99% homology with *Sus scrofa* stem cell growth factor receptor (kit) mRNA (accession no: FJ938289.1) confirming the expression of c-kit in juvenile and adult pig bladders in both the mucosa and detrusor.

### Histology and Immunohistochemistry

**Figure 5:**
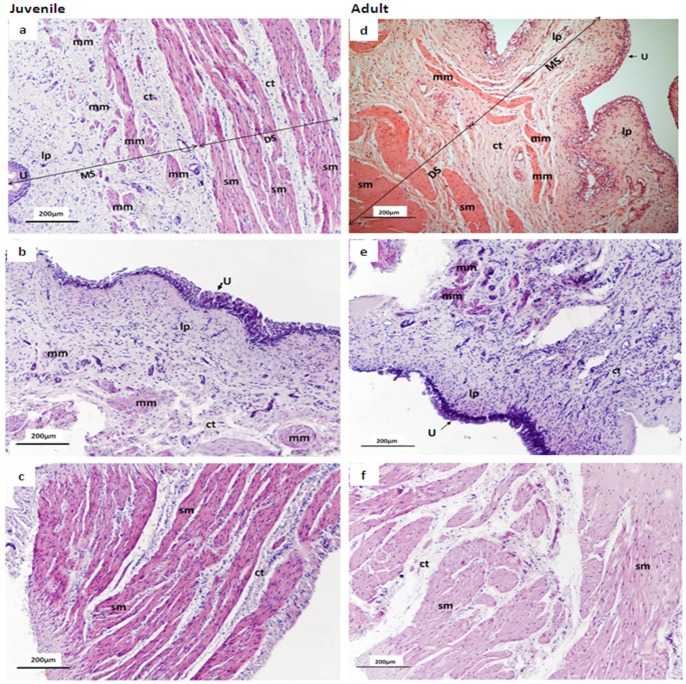
Low-power micrographs showing H&E staining of representative intact, mucosal and denuded-detrusor strips from juvenile and adult pig bladder dome. Left panel shows representative sections from juvenile bladder intact strip a) and mucosal strip (b) and denuded-detrusor strip (c); right panel shows an intact strip (d), a mucosal strip (e) and a denuded detrusor strip (f) from adult pig bladder. Note that the arrows in sections a) & d) demonstrate the plane of division between the mucosal and denuded-detrusor strips. MS, mucosal strip; DS, denuded-detrusor strip; sm, smooth muscle; ct, connective tissue; lp, lamina propria; U, urothelium; mm, muscularis mucosa.

Representative H&E stained sections of intact juvenile and adult pig bladders (detrusor+mucosa) are shown (Figures 5a and d). In both juvenile and adult mucosa, a discontinuous smooth muscle layer was observed, which we have termed the muscularis mucosa, analogous to that observed in human and guinea-pig urinary bladders [7]. Mucosal strips (Figures 5b and e) contained muscularis mucosa, with no apparent detrusor muscle present. The presence of minimal amounts of detrusor muscle cannot be entirely excluded. Denuded-detrusor strips (Figures 5c and f) were completely devoid of the mucosal layer (not illustrated).

**Figure 6:**
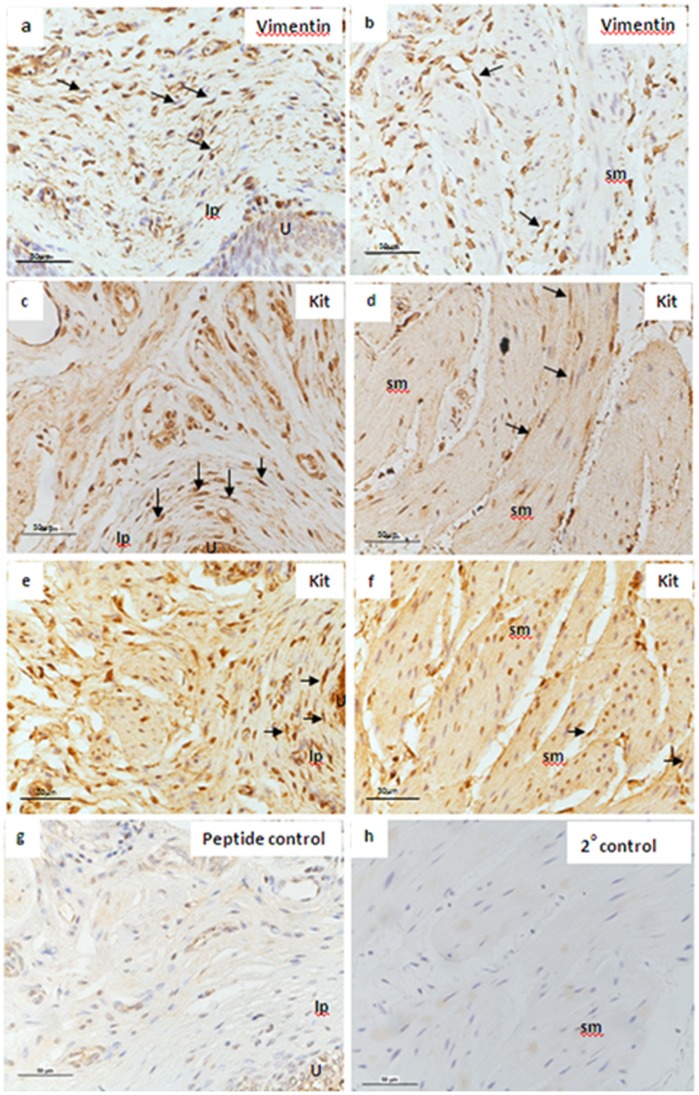
Confirmation of the expression of the tyrosine kinase receptor c-kit and vimentin in pig bladder using immunohistochemistry. Representative sections from paraffin-embedded mucosal and denuded-detrusor strips from adult and juvenile pig bladder; mucosal section showing vimentin-positive cells in the suburothelial and lamina propria region of the adult pig bladder (arrows) (a); vimentin-positive cells were also observed on the edge of and between smooth muscle bundles in denuded-detrusor sections in adult pig bladder (arrows) (b); mucosal section showing c-kit-positive spindle-shaped cells in the suburothelial and lamina propria region of the adult pig bladder (arrows) (c); c-kit positive cells were also observed on the edge and in between smooth muscle bundles (arrows) in adult pig bladder (d). A similar pattern of c-kit immunostaining was observed in sections of mucosa (e) and denuded-detrusor (f) from juvenile pig bladder. Negative control (c-kit peptide) (g) negative control (minus primary antibody) (h). sm, smooth muscle; lp, lamina propria; U, urothelium.

Immunostaining of mucosal sections from juvenile and adult pig bladders with antibodies directed at vimentin (Figure 6a) demonstrated vimentin-positive labelling throughout the mucosa, particularly within the suburothelial layer, similar to previous observations by Sadananda et al. [3]. Vimentin-positive cells were also observed on the edge of and between smooth muscle bundles in sections of denuded-detrusor from pig bladder (Figure 6b).

Immunostaining for c-kit subjectively was less widespread in distribution than for vimentin. c-kit immunopositive cells, with elongated cell bodies, were detected in the suburothelial layer of the adult and juvenile pig bladders (Figure 6c and e), and on the edge and between smooth muscle bundles (Figures 6d and f). Negative controls showed no immunostaining for c-kit (Figures 6g and h).

## Discussion

The contractile properties of the bladder mucosa have been previously described [3], [4] and it is speculated that phasic activity of intact bladder strips may be influenced by this layer [4], [5]. Although studies have shown that an intact mucosa is not required for development of phasic detrusor contraction in rabbit and pig bladders [19], [20], our data suggests that mucosa can contribute to this contractile activity in the region of the pig bladder dome. In the present study, mucosal strips from both juvenile and adult bladders demonstrated spontaneous contractions, consistent with previous reports demonstrating phasic contractile properties of urothelium and lamina propria from pig bladders [4]. In contrast to mucosal strips, the denuded-detrusor strips from juvenile and adult pig bladders did not demonstrate any basal phasic activity, and only in the presence of carbachol was phasic activity detected in this tissue. This suggests that intrinsic activity of the detrusor may originate and be organised from structures within the mucosal layer [21], though various explanations are possible. Urothelium is known to release transmitters, such as ACh, and these may be involved in generation or modulation of spontaneous contractions in the detrusor muscle [9]. In the present study, following stimulation with a low concentration of carbachol, denuded-detrusor strips from both juvenile and adult pig bladders demonstrated phasic activity. This suggests that phasic activity in the detrusor may be stimulated by the release of transmitters, although it is not possible to conclude which cell type they are acting on since muscarinic receptors are present on the detrusor, ICs and presynaptic nerve endings [22]. Although several groups have reported basal spontaneous activity in denuded-detrusor strips from pig bladder [4], [5], we were unable to detect this in the present study. Differences in the experimental and tissue preparation procedures may account for the discrepancies between the current and previous studies.

Interestingly, mucosal strips from juvenile pig bladders showed significantly greater amplitude of basal phasic activity vs. adult tissue. In contrast, the frequency of PCs in the mucosal strips from juvenile pig bladders was lower than in strips from adult bladders. High amplitude PCs seen in postnatal rat bladders may be necessary to promote maturation or organisation of the micturition reflex in neonates, when neural control of the bladder is immature [11]. In contrast to mucosal strips, there was no significant difference in either amplitude or the frequency of phasic activity in the presence of CCh between juvenile and adult tissues, which may suggest that the enhanced phasic activity detected in juvenile bladders may originate from the urothelium-suburothelium [9].

Structures within the bladder mucosa that potentially could mediate the phasic activity of this tissue include ICs and the muscularis mucosa [7]. ICs in the lamina propria demonstrate spontaneous depolarization and calcium transients, and although a pacemaker role has been suggested, this has yet to be confirmed [6]. The presence of c-kit positive ICs has previously been reported in the pig urinary tract, however, the exact location of these cells in the pig bladder was not clearly elucidated [23]. We confirm the expression of c-kit positive ICs in the lamina propria, and at the edge and between detrusor smooth muscle bundles in both juvenile and adult pig bladders. We also detected vimentin-positive cells in the lamina propria and the detrusor layers of the pig bladder, similar to a previous study [3]. Although not a selective IC marker, vimentin immunolabelling provides a useful means of visualising cell types which may include ICs. Thus, we speculate that a heterogenous population of ICs is present in the pig bladder, as seen in guinea-pig and human bladders [6].

The mechanism underlying the increased amplitude of PCs in mucosal strips from juvenile animals in the present study may be associated with a greater density or enhanced activity of ICs. In bladder tissues from patients with overactive bladder both enhanced numbers of ICs and increased sensitivity to imatinib are observed when compared to normal tissue [6]. In the present study, it was not possible to comment on differences between the number of ICs in the lamina propria of juvenile vs. adult pig bladders since we did not perform a full quantitative analysis. However, morphologically the bladders from both juvenile and adult pigs were similar in terms of immunostaining for vimentin and c-kit, and a congruent pattern of staining was observed in both groups of animals. The possible role of ICs in mediating the PCs of mucosa from juvenile and adult pigs was investigated using a tyrosine kinase receptor inhibitor, as previously described and used in bladder research to investigate the role of these cells [17], [24], [25], [26], [27]. Imatinib failed to inhibit PCs of mucosal strips from adult bladders and was only effective at its highest concentration (50 µM) in strips from juvenile bladders. Although there appeared to be a trend towards an increased sensitivity to imatinib in juvenile tissues, this was not statistically significant. Thus, it appears that differences in the activity of ICs may not be responsible for the differences observed between juvenile and adult tissues. However, this conclusion requires some caution, since not all ICs stain positive for c-kit and thus the population of c-kit positive cells may not play a role in phasic activity of the pig bladder, in contrast to other species [6]. In addition, the specificity of imatinib, particularly when used at high concentrations, has been questioned [28], [29], as acute application of imatinib *in vitro* is unlikely to suppress IC activity via inhibition of c-kit signalling. Instead imatinib may suppress phasic activity of smooth muscle by inhibition of intracellular calcium handling mechanisms [28]. In a recent study by Deng et al [26] it was demonstrated that ICs in rat bladder express T-type calcium channels (TTCCs) which may be responsible for the generation of calcium inward currents and spontaneous activity in these cells. Imatinib was able to decrease the inward calcium current in the bladder ICs but had no effect on the calcium currents detected in smooth muscle cells [26]. Conversely other groups have shown that relatively high concentrations of imatinib (50–100 µM) can suppress voltage dependent calcium channels (VDCCs), indicating a non-specific action [29]. In the present study, the potential inhibitory effect of imatinib on calcium handling was not investigated and therefore cannot be ruled out. In addition, imatinib also inhibits the platelet-derived growth factor (PDGFR) receptor kinase, alpha and beta [30]. A recent study in human and guinea-pig bladders has demonstrated that a subpopulation of c-kit positive ICs in the bladder also express PDGFR alpha [31]. It has also been demonstrated that ICs in the GI tract specifically express anoctamin 1 (ANO1), a type of calcium activated chloride channel [32]. Even though we are yet to demonstrate the expression of PDGFR-alpha in the pig bladder, we were able to detect the expression of ANO1 mRNA in the mucosa and detrusor layers in pig bladder [33]. Although the exact location of these channels is to be determined, the acute effects of imatinib on bladder phasic activity may also be attributed to inhibition of PDGFR alpha receptors/ANO1 channels on ICs, although the role of PDGFR alpha receptor signalling in functional activity of ICs is unknown.

In the present study, following stimulation with a low concentration of CCh, denuded-detrusor strips from both juvenile and adult pigs demonstrated PCs, although there was no significant difference in the amplitude or the frequency of these PCs between juvenile and adult tissues. Muscarinic receptor stimulation by application of CCh to whole-bladder sheets *in situ* preparations can increase the frequency of calcium-transients in detrusor ICs [34]. Imatinib (10 & 50 µM) was able to reduce the amplitude of cholinergic-induced PCs in both juvenile and adult pig denuded-detrusor strips, although juvenile tissues demonstrated a trend towards being more sensitive to this inhibition than the adult tissues. Although this possibly points to potential differences in the population or activity of ICs in juvenile pig bladder, the same caution must be applied with regard to possible non-specific effects of imatinib on calcium channels at the higher concentration (50 µM) and the fact that not all ICs are c-kit positive.

In addition to ICs, a pharmacologically and physiologically distinct type of smooth muscle, termed the muscularis mucosa, has been described in the lamina propria between the basement membrane of the urothelium and detrusor [7]. Using H&E staining we were able to detect a similar distinct layer of smooth muscle in the mucosal layer of the pig bladder, similar to the muscularis mucosa found in the guinea-pig urinary bladder [7]. In the guinea pig bladder the muscularis mucosa exhibits spontaneous Ca^2+^ transients which have been suggested to underlie phasic contractions [7]. Whether this layer has a similar role in the pig bladder is not yet clear, but it may underlie the phasic activity and contractile responses observed in isolated mucosal strips in the present study and those previously demonstrated [3], [4]. Thus, the differences observed in phasic activity in mucosal strips from juvenile pig bladder versus adult pig bladders could be due to alterations at the level of the muscularis mucosa, which requires further investigation.

In conclusion, mucosal strips from juvenile and adult pig bladders demonstrated basal PCs, which was absent in denuded-detrusor strips, suggesting that in the pig bladder dome intrinsic activity may be driven by various structures in the mucosal layer. We were able to detect c-kit-positive and vimentin-positive ICs in the lamina propria and detrusor layers of the juvenile and adult pig urinary bladder. Mucosal strips from juvenile pigs showed increased basal PCs when compared to strips from adult bladders, which may be a result of enhanced activity of ICs in the lamina propria or differences in the functional properties of the muscularis mucosa in the mucosal layer compared to adult bladders. Imatinib inhibited basal PCs of mucosal strips from juvenile bladders, and cholinergic-induced phasic activity of denuded-detrusor strips from juvenile and adult bladders, though only at the highest concentrations.
